# C-Terminal Auto-Regulatory Motif of Hepatitis C Virus NS5B Interacts with Human VAPB-MSP to Form a Dynamic Replication Complex

**DOI:** 10.1371/journal.pone.0147278

**Published:** 2016-01-19

**Authors:** Garvita Gupta, Jianxing Song

**Affiliations:** Department of Biological Sciences, Faculty of Science, National University of Singapore, Singapore, Singapore; Scripps Research Institute, UNITED STATES

## Abstract

Hepatitis C virus (HCV) is a pathogen of global importance and nearly 200 million people are chronically infected with HCV. HCV is an enveloped single-stranded RNA virus, which is characteristic of the formation of the host membrane associated replication complex. Previous functional studies have already established that the human ER-anchored VAPB protein acts as a host factor to form a complex with HCV NS5A and NS5B, which may be established as a drug target. However, there is lacking of biophysical characterization of the structures and interfaces of the complex, partly due to the dynamic nature of the complex formation and dissociation, which is extensively involved in intrinsically-disordered domains. Here by an integrated use of domain dissection and NMR spectroscopy, for the first time we have successfully deciphered that the HCV NS5B utilizes its auto-regulatory C-linker to bind the VAPB-MSP domain to form a dynamic complex. This finding implies that the NS5B C-linker is capable of playing dual roles by a switch between the folded and disordered states. Interestingly, our previous and present studies together reveal that both HCV NS5A and NS5B bind to the MSP domains of the dimeric VAP with significantly overlapped interfaces and similar affinities. The identification that EphA2 and EphA5 bind to the MSP domain with higher affinity than EphA4 provides a biophysical basis for further exploring whether other than inducing ALS-like syndrome, the HCV infection might also trigger pathogenesis associated with signalling pathways mediated by EphA2 and EphA5.

## Introduction

Hepatitis C virus (HCV), first discovered in 1989 [1], is a pathogen of global importance due to significant health problems throughout the world [1–3]. Nearly 200 million people are chronically infected with HCV who are at risk of developing liver disease including liver cirrhosis and hepatocellular carcinoma. HCV is an enveloped single-stranded RNA virus, which belongs to the *Hepacivirus* genus in the *Flaviridae* family. The genome of HCV is positive sense single ~9.6 kb long RNA and encodes ~3000 residue polyprotein, which is subsequently processed by viral and cellular proteases into 10 mature structural and nonstructural regulatory proteins on the rough endoplasmic reticulum (ER). Nonstructural proteins, which coordinate the intracellular processes of the virus life cycle, include P7 ion channel, NS2 protease, NS3 serine protease and helicase, NS4A, NS4B, NS5A proteins and NS5B RNA-dependent RNA polymerase (RdRp) [4].

HCV infection induces distinct alteration of membranes which form sponge-like inclusions, called as “membranous webs”. Studies have suggested that these membranous webs constitute the sites for RNA replication, on which replication proteins and viral RNA localize [5–7]. On the membrane-associated replication machinery, RNA synthesis is catalysed by the viral RdRp activity of NS5B facilitated by both viral NS proteins and human cell host factors [8, 9]. Although viral NS proteins, RNA are main components of replication complex, the exact composition and details of replication machinery is poorly understood. Like all viruses, HCV infection acts to reprogram the cellular metabolism in such a way that infected cells devote themselves to orchestrating the production of new viruses. During this process, HCV heavily relies on human cell host factors for its replication. Thus, there is of significant interest in identifying those host factors which may be established as critical targets for drug design.

**Fig 1:**
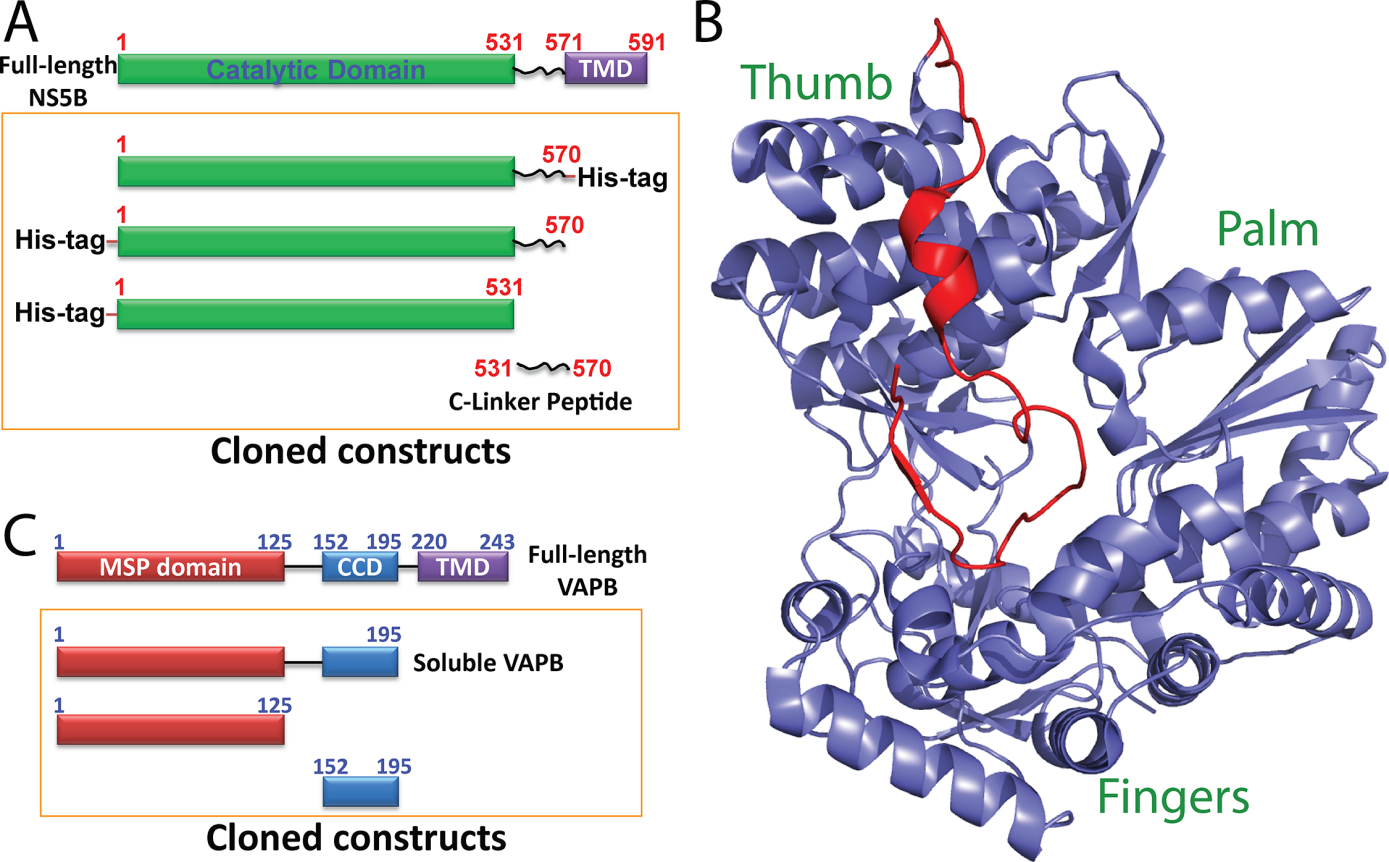
Domain organization and structure of HCV NS5B and human VAPB. A. Domain organization of HCV NS5B and differentially dissected proteins used in the present study. B. Crystal structure of NS5B(1–570) (PDB code of 1C2P) with the C-linker over residues 531–570 colored in red. C. Domain organization of human VAPB and differentially dissected proteins used in the present study.

Recently, NS5B, the core component of HCV replication complex, has emerged as a key target for the development of small molecules that can selectively inhibit the replication as mammalian cells lack an RdRp equivalent. NS5B contains 591 residues, in which the catalytic domain over residues 1–531 is connected to a membrane inserting sequence 571–591 with a linker over residues 531–570 (Fig 1A). In 1999, the structures of NS5B have been determined, whose catalytic domain is composed of three subdomains (Fig 1B): palm domain which harbors the active site, finger domain and thumb domain [10–12]. Further determination of the NS5B structures together with C-linker deciphered that unlike other RdRp, the HCV NS5B has a close conformation of the active site [13–15], in which the C-linker folds back into the active center and thus regulates the enzymatic activity (Fig 1B). This unique feature appears to play a key role in RNA synthesis initiation [12–16]. Very recently, the structural changes of NS5B required for the HCV RNA replication has been successfully delineated: the thumb domain β-loop and C-linker, which is buried within the encircled active-site cavity (apo-state), vacate the active-site cavity and generate a larger cavity (open form) for RNA elongation [13–15]. As such, the C-linker acts as an auto-regulatory motif for the HCV NS5B polymerase [14–19].

Due to a high rate of mutation for viral proteins during the life cycle, targeting host-viral protein interaction interfaces for development of antiviral drugs has become increasingly attractive in recent years. Previous functional studies have identified the involvement of human vesicle-associated membrane protein-associated proteins (VAPs) in HCV replication through interaction with NS5A and NS5B [20–23]. Briefly, human VAPB protein has been biologically established by two groups to interact with both HCV NS5A and NS5B to form the HCV replication complex [20,22,23], but the high-resolution structure remains undetermined for the NS5B-VAPB interaction.

Therefore, delineation of high-resolution structures and interfaces of the VAPs-NS5A/NS5B interactions represents an essential step to facilitate further drug development by targeting their interfaces. The VAP family includes VAPA, VAPB and VAPC, out of which VAPB is composed of three domains (Fig 1C): an N-terminal nematode major sperm protein (MSP) domain, a central coiled-coil domain (CCD) and a C-terminal transmembrane domain (24–26). Previously, by high-resolution NMR characterization of structures and interactions of the differentially-dissected domains of HCV NS5A and human VAPB proteins, we have successfully deciphered that the intrinsically unstructured domain 3 of HCV NS5A forms a "fuzzy” or dynamic complex with the MSP domain of VAPB [26]. Interestingly, the mutation sites within the MSP domain associated with amyotrophic lateral sclerosis (ALS) have been shown to located on the NS5A-MSP interaction interface [25–27]. On the other hand, the MSP domain has been identified to be an endogenous ligand for Eph receptors [27,28], which consists of 16 members and represents the largest transmembrane tyrosine kinase receptors [29]. Strikingly, we revealed that the interface on the MSP domain for interacting with NS5A is also overlapped with that used for binding to EphA4 [26], the only known ALS modifier [30,31], thus rationalizing a potential linkage between HCV infection and ALS pathogenesis [26,32].

In the present study, by the same approach [26,33], we aimed to NMR characterize and quantify the interactions of human VAPB with HCV NS5B as well as 6 Eph receptors. Remarkably, the results reveal that the isolated NS5B C-linker is intrinsically disordered in solution. Nevertheless, it is capable of binding the human VAPB-MSP domain over surfaces which have significant overlaps with those for binding HCV NS5A and human Eph receptors.

## Results

### Generation and characterization of differently dissected NS5B proteins

To study the interaction between NS5B and VAPB, we have dissected both VAPB and NS5B into different combinations of domains. For human VAPB, here we used the same dissected proteins which we previously cloned and characterized for mapping the NS5A-VAPB interactions [26], namely the soluble VAPB only with the transmembrane domain (TMD) deleted, the MSP domain and coiled-coil domain (CCD) (Fig 1C). For HCV NS5B, as it has been previously demonstrated that the removal of C-terminal membrane-interacting residues (571–591) is required to enhance the solubility but retains the similar activity of polymerase [12–14], here we cloned and expressed four dissected proteins: namely NS5B(1–570) with either N- or C-terminal His-tags, NS5B(1–531) only containing the catalytic domain [11], as well as the C-linker peptide containing residues 531–570 (Fig 1A).

All VAPB recombinant proteins were expressed and purified by the same procedures as previously described [26]. Three recombined NS5B proteins with His-tag were successfully expressed in BL21 cells and subsequently purified with Ni^2+^-affinity chromatography followed by Heparin affinity chromatography. On the other hand, the C-linker peptide was cloned into pGEX-4T1 vector with GST-tag. The GST-fused peptide was first purified by affinity chromatography with glutathione-Sepharose 4B beads, and subsequently cleaved to release the C-linker peptide which was purified by reverse-phase (RP) C18 column using HPLC system.

**Fig 2:**
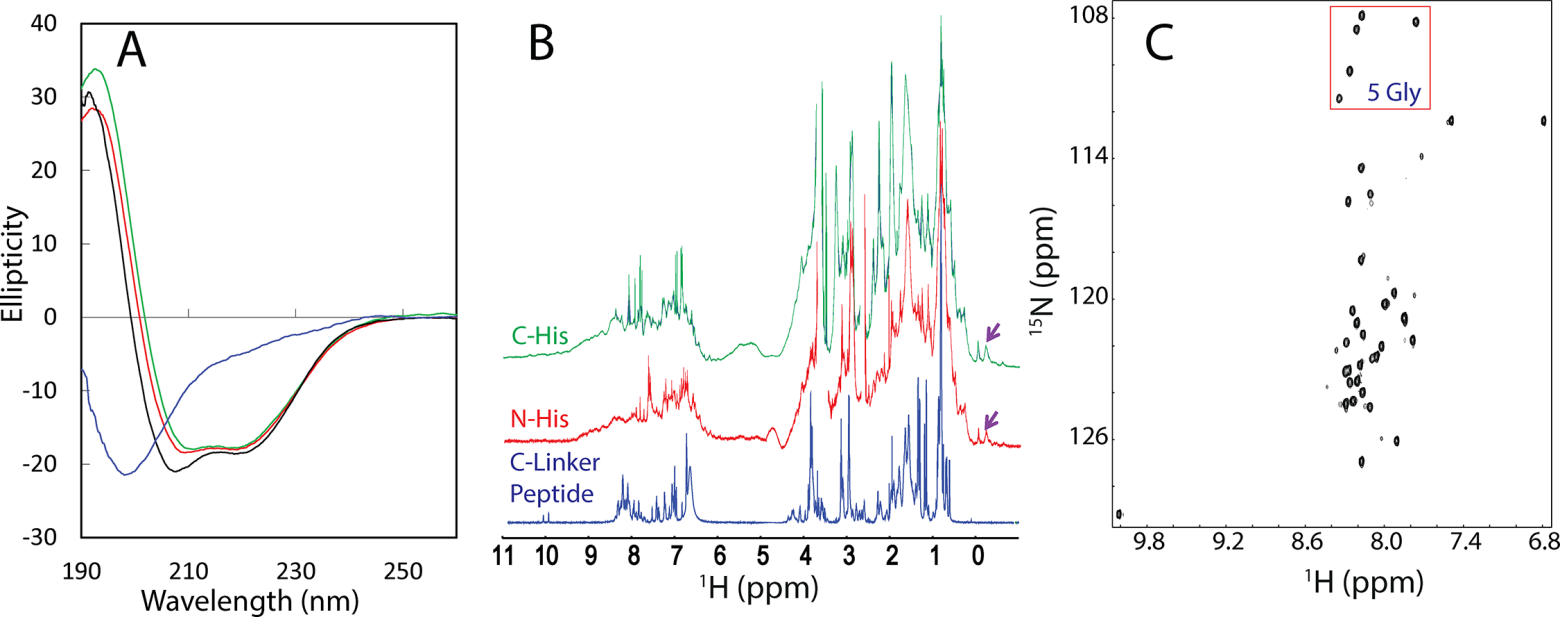
CD and NMR characterization of HCV NS5B proteins. A. Far UV CD spectra of C-His-tagged NS5B(1–570) (green), N-His-tagged NS5B(1–570) (red), N-His-tagged NS5B(1–531) and NS5B C-linker (531–570) (blue). B. One-dimensional ^1^H NMR spectra of NS5B proteins. Purple arrows are used to indicate several up-field NMR peaks characteristic of a well-folded protein. C. ^1^H-^15^N HMR HSQC spectrum of the ^15^N-labeled NS5B C-linker (531–570). The red box is used to indicate HSQC peaks of 5 Gly residues in the C-linker peptide.

As shown in Fig 2A, the NS5B(1–570) proteins with the N- and C-terminal His-tag respectively have the similar far-UV CD spectra characteristic of a well-folded helix-dominant protein, with two negative signals at ~209 and 222 nm, as well as a very large positive signal at ~193 nm, consistent with the well-defined crystal structures previously determined [10–15]. The NS5B(1–531) protein, despite having some minor difference from those of NS5B(1–570) proteins, also has a far-UV CD spectra characteristic of a well-folded helix-dominant protein. In fact, comparison of the crystal structures of NS5B(1–570) [15] and NS5B(1–531) [10,11] reveals that the catalytic domain have almost the same structures in two constructs. Consistent with CD results, their ^1^H one-dimensional NMR spectra of NS5B(1–570) proteins are also similar, both of which have several very up-field peaks (Fig 2B), clearly suggesting that they are well-folded with the tight tertiary packing. Unfortunately, due to the short transverse relaxation time (T2) resulting from the very large molecular size, the NMR resonance peaks are very broad particularly over the amide proton region (Fig 2B). Consequently, no high-quality ^1^H-^15^N NMR HSQC spectrum could be acquired.

By contrast, as shown in Fig 2A, the C-linker peptide has a far-UV CD spectrum typical of a highly-disordered protein with the maximal negative signal at 198 nm, and lacking any positive signal at 190 nm. Furthermore, very up-field peaks are totally absent in the ^1^H one-dimensional NMR spectrum (Fig 2B), clearly indicative of the lack of any tight tertiary packing. Its ^1^H-^15^N NMR HSQC spectrum (Fig 2C) also indicated that it lacks any tight tertiary packing as judged from very narrow ^1^H (~0.86 ppm) and ^15^N (~19 ppm) spectral dispersions. More specifically, the peptide contains 40 residues plus two extra N-terminal Gly-Ser residues from the cleavage of the GST-fusion protein, in which there are 6 Gly and 3 Pro residues. As the HSQC peak of the N-terminal Gly is usually undetectable due to the fast exchange of its amide protons with water, and 3 Pro residues have no amide proteins, there should be 38 backbone HSQC peaks in the HSQC spectrum. However, although HSQC peaks of 5 Gly residues within the NS5B C-linker peptide are all detectable and well-separated (Fig 2C), only ~32 strong HSQC peaks could be detected for backbone amides (Fig 2C). This observation implies that there are 6 residues which might have their HSQC peaks overlapped with other; or/and become very weak or even undetectable due to their fast exchange with water, or/and due to conformational exchanges on μs-ms time scale. Therefore, as judged by its CD, 1D and HSQC spectra, the isolated C-linker appears to be predominantly disordered in solution, lacking of any well-formed secondary structures and tight tertiary packing. Unfortunately, due to its aggregation at high concentration, we attempted but failed to collect high-quality triple-resonance NMR experiments to achieve its assignments.

### Identification of the binding interfaces on VAPB and NS5B

**Fig 3:**
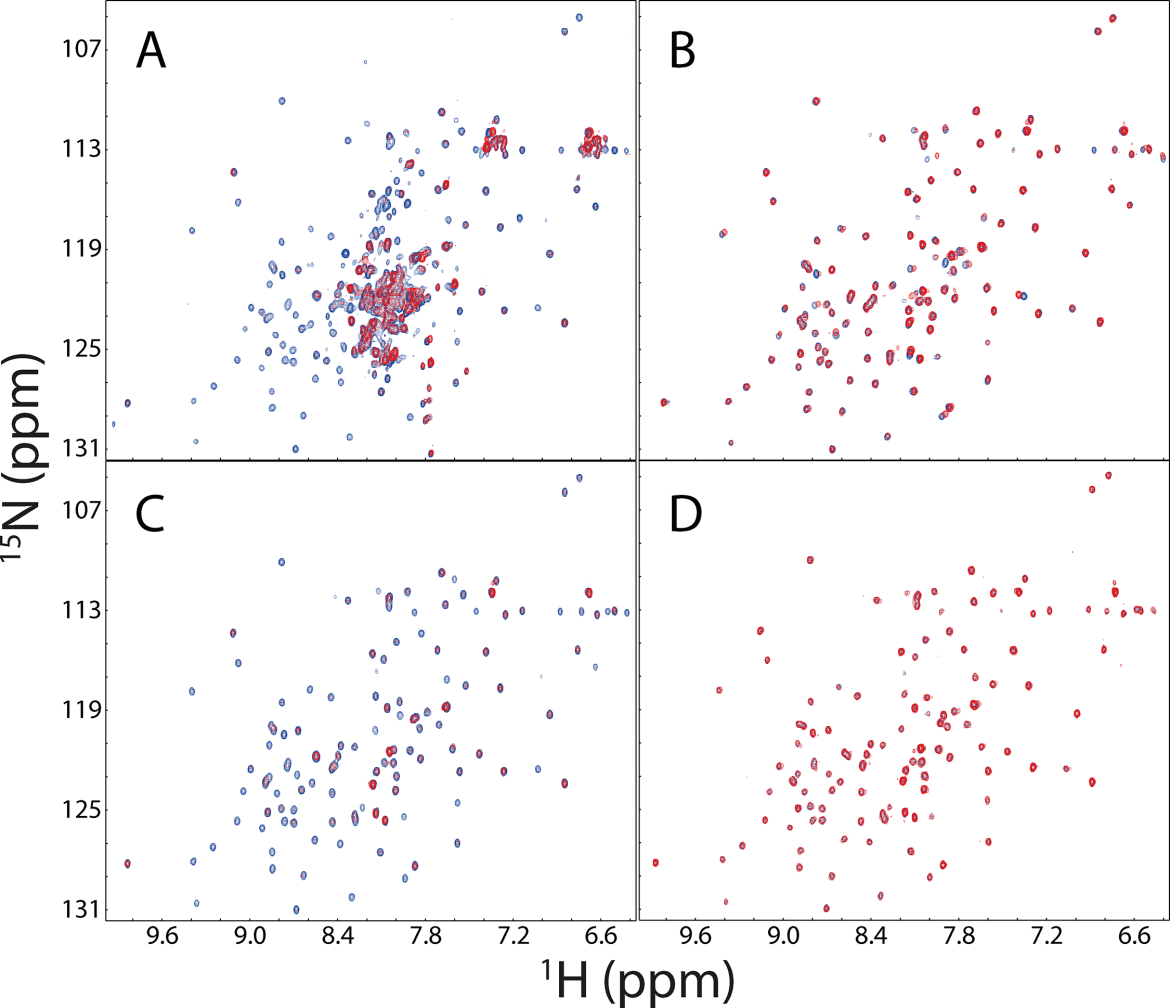
HSQC characterization of the binding between VAPB and NS5B. A. ^1^H-^15^N HSQC spectra of the ^15^N-labeled soluble VAPB protein (1–195) in the absence (blue) and in the presence of the unlabelled N-His-tagged NS5B(1–570) at a molar ratio of 1:2 (red). In the presence of the unlabelled N-His-tagged NS5B(1–570), most well-dispersed peaks disappeared which were identified to be from the MSP domain while the remaining peaks (red) are from the coiled coil domain, based on our NMR assignments of the human VAPB domains previously published (25–27). B. ^1^H-^15^N HSQC spectra of the ^15^N-labeled VAPB-MSP domain in the absence (blue) and in the presence of the unlabelled C-His-tagged NS5B(1–570) at a molar ratio of 1:4 (red). C. ^1^H-^15^N HSQC spectra of the ^15^N-labeled MSP domain in the absence (blue) and in the presence of the unlabelled N-His-tagged NS5B(1–570) at a molar ratio of 1:2 (red). D. ^1^H-^15^N HSQC spectra of the ^15^N-labeled MSP domain in the absence (blue) and in the presence of the unlabelled N-His-tagged NS5B(1–531) at a molar ratio of 1:4 (red).

Previously, we have cloned and shown that the NS5B(1–570) with a C-terminal His-tag was activity in binding the human VAPC protein [33] and DDX5 [34]. So here we first tested its binding to the soluble full-length VAPB. However, the NMR titrations indicated a lack of significant interaction between them, disagreeing with the previous functional results that HCV NS5B interacts with human VAPB protein to form the replication complex [20,22,23]. This led us to suspecting that the binding region of NS5B to VAPB is mostly located on the C-terminus and therefore the presence of the C-terminal His-tag would block the binding, possibly because the His-tag at the C-terminus may be incompatible with interacting with the MSP domain, or/and alter the dynamics of the C-linker because a comparison of the NS5B (1–570) structures with the His-tag at the N-terminus (PDB code of 1C2P) [11] or at the C-terminus (PDB code of 1QUV) [12] indicates that the C-terminal linker adopts a similar structure in both crystal structures. Therefore, we then cloned the NS5B(1–570) with a N-terminal His-tag and characterized its binding to the soluble VAPB protein. Indeed, as shown in Fig 3A, the addition of the unlabelled N-His-tagged NS5B(1–570) induced a disappearance of many HSQC peaks of the ^15^N-labeled VAPB, clearly indicating the significant binding between two proteins. With NMR assignments of the VAPB domains we previously published [25–27], we thus identified that the disappeared peaks are almost all from the MSP domain of VAPB, while the remaining peaks (red) are from the coiled-coil domain (CCD) of VAPB. We also titrated the ^15^N-labeled CCD of VAPB by both NS5B(1–570) proteins but found no significant perturbation. These results clearly reveal that HCV NS5B binds to the MSP domain of human VAPB protein, completely consistent with previous biological studies [20,23,24].

To identify the region of HCV NS5B which binds the MSP domain, we conducted detailed HSQC titrations of the ^15^N-labeled MSP domain by different NS5B proteins. The C-His-tagged NS5B(1–570) only induced slight shifts of several HSQC peaks of the MSP domain (Fig 3B), while the N-His-tagged NS5B(1–570) triggered a dramatic disappearance of the majority of the peaks (Fig 3C). Most strikingly, the NS5B(1–531) containing only the catalytic domain shows almost no perturbation on the HSQC peaks of the MSP domain. These results together decipher that the C-linker of the HCV NS5B plays a central role in binding the MSP domain of the human VAPB.

### Quantification of the binding between NS5B C-linker and MSP domain

**Fig 4:**
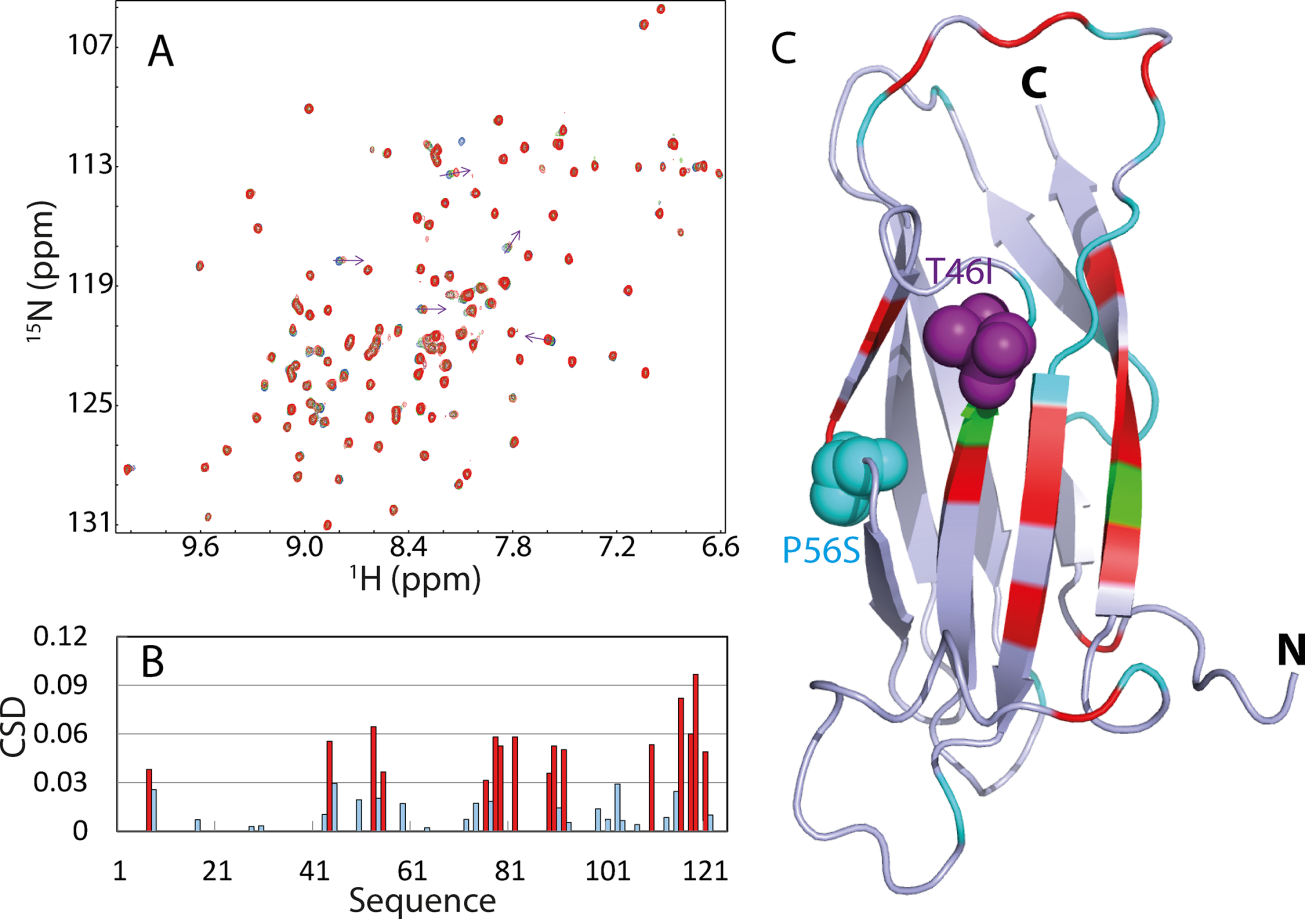
Quantification of the binding between MSP domain and NS5B C-linker. A. ^1^H-^15^N HSQC spectra of the ^15^N-labeled MSP domain in the absence (blue) and in the presence of the unlabelled NS5B C-linker (531–570) at a molar ratio of 1:1 (green) and 1:3 (red). B. Residue-specific chemical shift difference (CSD) of the MSP domain in the presence of the NS5B C-linker (531–570) at a molar ratio of 1:3. The bars for the residues with the CSD value > 0.03 (average + STD) are colored in red. C. Crystal structure of the MSP domain we previously determined in which cyan is used for indicating Pro and residues with missing HSQC peaks in the free state; green for residues disappeared in the presence of the NS5B C-linker (531–570), and red for the residues with the CSD value > 0.03 at a molar ratio of 1:3. The pink and cyan spheres are used to indicate the sites of the mutations Thr46 (to Ile) and Pro56 (to Ser) respectively, which are associated with familial ALS.

We thus generated the recombinant NS5B C-linker peptide which is highly disordered in solution (Fig 2). However, it is active in binding the MSP domain. As shown in Fig 4A, a gradual addition of the peptide induces both shifts and intensity reductions of a set of HSQC peaks of the ^15^N-labeled MSP domain. As the peptide started to show visible aggregation at high concentration, we were only able to titrate the MSP domain with the peptide at the molar ratio of 1:3 (MSP:peptide) at which the shifts of the peaks are almost saturated. However, the C-linker peptide alone appears to have less ability to provoke the disappearance of the MSP peaks than the N-His-tagged NS5B(1–570), probably because the presence of the very large catalytic domain might provide additional binding residues, or/and trigger radical dynamics of the MSP domain on μs-ms time scale.

Fig 4B presents the chemical shift difference (CSD) of the HSQC peaks for the MSP domain at 1:3 (MSP:peptide), while Fig 4C shows the MSP structure we previously determined [25], whose residues are colored if their HSQC peaks disappeared (Thr46, Ser117), or significantly shifted with the CSD value > 0.03 (average + STD). Interestingly, these residues are all located on one side of the MSP structure (Fig 4C). Most strikingly, two ALS-causing mutations Pro56Ser and Thr46Ile are also located on this side and in particular the HSQC peaks of Thr46 disappeared upon binding to the NS5B C-linker. Remarkably, the binding interface of the MSP domain to the NS5B C-linker is highly overlapped with that for binding the NS5A Domain 3 as we previously identified by NMR [26].

With the well-established procedure to obtain the dissociation constants (Kd) by fitting the data of HSQC titrations, which we have previously utilized to quantify the NS5A-MSP [26] and NS5B-VAPC [33] interactions, here we gain the residue-specific Kd values for significantly-shifted residues, with an average of 8.8 μM (Table 1). It is worth to note that this value is highly similar to that (8.0 μM) for the binding of the MSP domain to the HCV NS5A D3 domain [26]. The results here hence reveal that the HCV NS5A and NS5B bind to the highly overlapped interfaces of the human MSP domain with very similar affinities.

**Table 1:** Dissociation constants for the binding of the MSP domain with NS5B C-linker, EphA2 and EphA5 obtained by fitting NMR titration data.

	NS5B C-Tail	EphA2	EphA5
Residue	Kd (μM) ± SE	Kd (μM) ± SE	Kd (μM) ± SE
6Q		7.9 ± 0.6	
44V		4.1 ± 1.2	36.2 ± 2.8
53C	1.4 ± 0.3	6.3 ± 0.3	57.1 ± 0.2
76F			59.2 ± 3.4
78Y		10.1 ± 1.1	89.2 ± 8.9
79D	13.6 ± 1.1	18.9 ±1.6	77.9 ± 4.2
82E	12.8 ± 0.9		
90V	9.0 ± 1.2		53.8 ± 1.1
91Q			74.6 ± 5.0
92S			81.5 ± 4.9
116D	5.7 ± 0.7		
118K	2.9 ± 0.6		
119L	16.0 ± 2.8		
122V		14.2 ± 0.7	
**Average**	**8.8 ± 1.1**	**10.3 ± 0.9**	**66.2 ± 3.8**

### Characterization of the binding of the MSP domain with Eph receptors

Previously, the human MSP domain has been biologically identified to be cleaved and subsequently secreted to serve as a ligand of the ligand binding domain (LBD) of Eph receptors, and furthermore it was shown that the interaction between EphA4 and VAPB-MSP domain is involved in ALS [28,30,31]. However, so far the high-resolution biophysical characterization has been conducted only on the MSP-EphA4 interaction previously by us [26,27]. Here we further extended to characterizing the interactions of the MSP domain to EphA2, EphA5, EphA6, EphA7 and EphA8. These Eph LBDs were expressed and purified by Ni^2+^-NTA affinity chromatography followed by FPLC size exclusion chromatography as we previously described [26,27,31,35–38].

**Fig 5:**
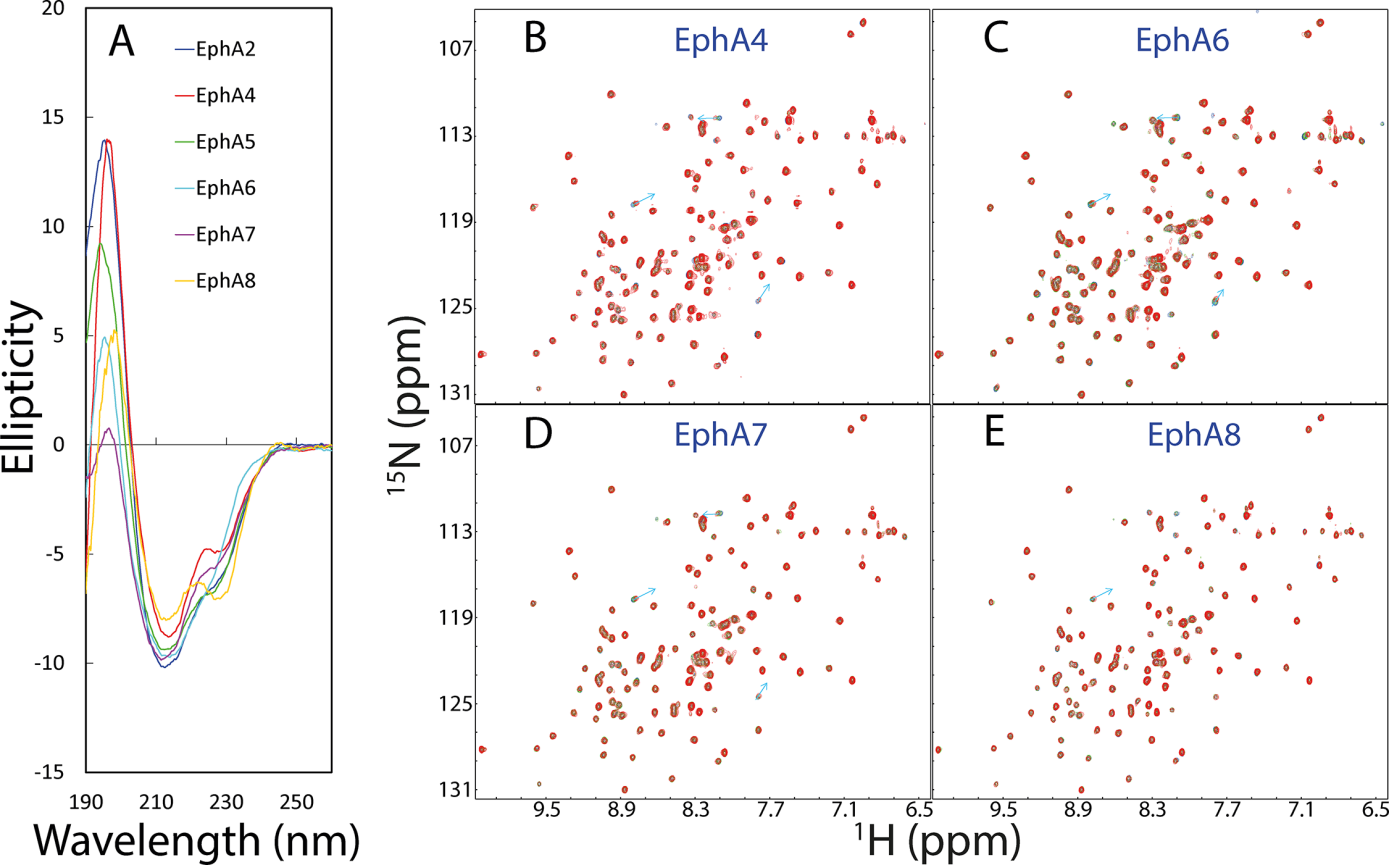
Characterization of the binding between MSP and Eph LBDs. A. Far-UV CD spectra of 6 Eph LBDs. ^1^H-^15^N HSQC spectra of the ^15^N-labeled MSP domain in the absence (blue) and in the presence of the unlabelled LBDs of EphA4 (B), EphA6 (C), EphA7 (D) and EphA8 (E) at a molar ratio of 1:1 (green) and 1:4 (red).

As shown in Fig 5A, all 6 Eph LBDs are well-folded as judged from their far-UV CD spectra. Subsequently, we titrated the ^15^N-labeled MSP domain with the unlabelled Eph LBDs. As previously we have extensively characterized the interaction of the MSP domain with EphA4 LBD by ITC, NMR and modelling [27], here we used it as a reference. Exactly as we have previously shown [27], only very minor shifts of several HSQC peaks could be observed for the ^15^N-labeled MSP domain (Fig 5B), upon adding the unlabelled EphA4 LBD at a molar ratio of 1:4 (MSP:Eph4). Similar scenarios are also observed for EphA6 (Fig 5C), EphA7 (Fig 5D) and EphA8 (Fig 5E). This implies that the binding affinities for the MSP domain to EphA6, EphA7 and EphA8 are similar to or even weaker than that to EphA4.

**Fig 6:**
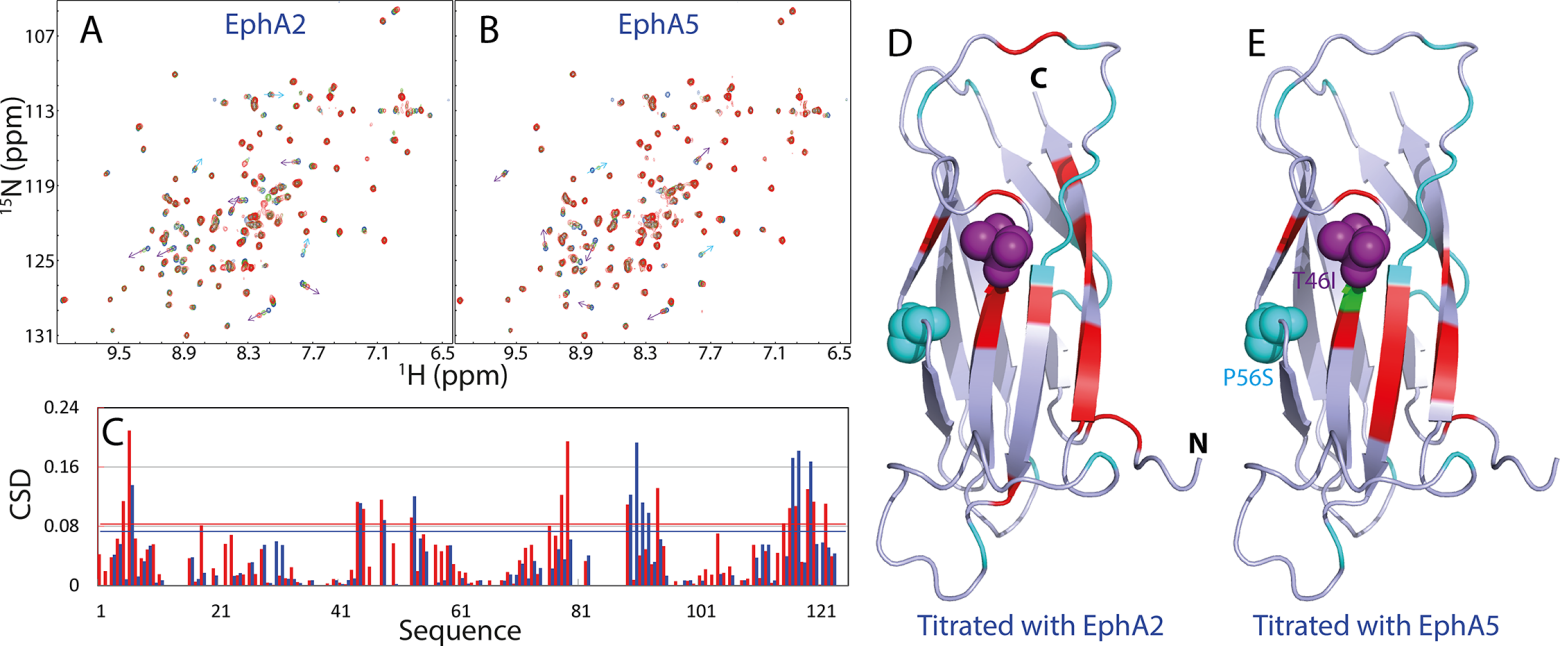
Quantification of the binding between MSP domain and EphA2/EphA5. ^1^H-^15^N HSQC spectra of the ^15^N-labeled MSP domain in the absence (blue) and in the presence of the unlabelled LBDs of EphA2 (A) and EphA5 (B) at a molar ratio of 1:1 (green) and 1:3 (red). C. Residue-specific chemical shift difference (CSD) of the MSP domain in the presence of EphA2 (red) and EphA5 (blue) at a molar ratio of 1:4. Crystal structure of the MSP domain in which cyan is used for indicating Pro and residues with missing HSQC peaks in the free state; green for residues disappeared in the presence of EphA2 (D) or EphA5 (E), and red for the residues with the CSD value > 0.083 for EphA2 and >0.073 for EphA5 at a molar ratio of 1:4. The pink and cyan spheres are used to indicate the sites of the mutations Thr46 (to Ile) and Pro56 (to Ser) respectively, which are associated with familial ALS.

On the other hand, the HSQC titrations reveal that EphA2 (Fig 6A) and EphA5 (Fig 6B) LBDs are able to trigger both significant shift and intensity reduction for many HSQC peaks of the ^15^N-labeled MSP domain at low molar ratios, and particularly the peak shifts have become mostly saturated for EphA2 LBD at 1:4 (MSP:EphA2) and for EphA5 at 1:6 (MSP:EphA2). Fig 6C presents the chemical shift differences (CSD) of the MSP HSQC peaks in the presence of EphA2 and EphA5 at 1:4 (MSP:Eph), while Fig 6D and 6E show the MSP structures, whose residues are colored if their HSQC peaks disappeared, or significantly shifted with the CSD value > 0.083 for EphA2 and 0.073 for EphA5 (average + STD). Interestingly, the significantly perturbed residues triggered by binding to EphA2 and EphA5 are located on the same side of the MSP structure (Fig 4C), which is also highly similar to those by the NS5B C-linker (Fig 4C), and by EphA4 [27].

We also obtained the Kd values for significantly shifted residues induced by binding with EphA2 and EphA5, which are presented in Table 1. The average Kd value is 10.3 μM for the MSP-EphA2 interaction while is 66.2 μM for the MSP-EphA5. This suggests that EphA2 LBD binds to the MSP with the highest affinity among 6 Eph LBDs investigated here. In the future, we are also interested in characterizing the binding affinity of the MSP domain with the rest of Eph receptors.

## Discussion

RNA viruses such as HCV are characteristic of the formation of the host membrane associated replication complexes [20–23,39,40]. Although it is widely anticipated that studies of RNA virus replication machineries will have a critical impact on antiviral drug development because of their essential and specific roles in virus replication, currently high-resolution structures and interaction interfaces remain relatively unknown for the HCV replication complexes [41]. Previously, it has been biologically established that both HCV NS5A and NS5B interact with the human VAPB proteins to constitute the core components of the HCV replication complex [20–23] but so far there had been lacking of extensive atomic-resolution structural and binding characterization. One challenge for the biophysical studies is associated with the dynamic nature of the complex formation and dissociation, with extensive involvement of intrinsically disordered domains/regions. Although the dynamics of the replication complex play a central role in implementing its functions, this makes it very challenging for crystallographic investigations. In this regard, NMR spectroscopy represents the only powerful tool to delineate the weak binding involved in intrinsically disordered domains at atomic resolution [26, 42–48]. Indeed, previously we have conducted a systematic investigation on the NS5A-VAPB interactions by dissecting both VAPB and NS5A proteins into 14 domains/fragments, followed by extensive structural and binding characterizations with heteronuclear NMR spectroscopy. Our study successfully reveals that the intrinsically disordered Domain 3 of NS5A is responsible for dynamically binding to the MSP domain of VAPB [26].

In the present study, by use of the domain dissection followed by NMR characterization, for the first time we have successfully deciphered that the HCV NS5B binds to the MSP domain of the human VAPB mostly by its C-linker. This finding is interesting as in the NS5B(1–570) structure with a close conformation of the active site [13–15], the C-linker folds back into the active center of the catalytic domain (Fig 1B), which thus acts to auto-regulate the RdRp enzymatic activity [13–15,49]. The regulatory role of the C-linker in mediating catalysis is also supported by previous functional studies, which showed that the enzymatic activity of the C-terminally truncated NS5B(1–531) could be up to ~150-fold higher than that of NS5B(1–570) [13,14,49–51]. Interestingly, the C-linker could be clearly visualized in crystal structures [13–15], which is composed of a short helix and β-strand despite also having regions without regular secondary structures. As such, it is expected that in the context of NS5B(1–570) having the catalytic domain, the C-linker would have less dynamic secondary structures and tighter packing than what observed on the isolated C-linker in solution, which has no stable secondary structure and tight packing as clearly indicated by CD and NMR. As such, our study suggests that the C-linker is able to play dual roles by a conformational and dynamic transformation between the folded and disordered states, very similar to what we previously observed on the auto-inhibitory domain of PAK4 kinase [48]. Remarkably, our study also reveals that the HCV NS5B C-linker and NS5A domain 3 bind the human VAPB MSP domain, with the similar affinities and interfaces on the MSP domain [26]. The high dynamics required for formation and dissociation of this replication complex appears to be nicely achieved by the relative-weak affinity of the interactions of HCV NS5A/NS5B to the human MSP domain, which is made possible by extensive involvement of intrinsically disordered HCV domains including NS5A Domain 3 and NS5B C-linker.

Previously the MSP domain has been functionally identified to act as an endogenous inhibitor for the ephrin-binding domain of Eph receptor family [28], which is composed of 10 EphA and 6 EphB members with a large binding promiscuity [29]. On the other hand, P56S and T46I mutations disrupting the MSP fold have been shown to cause ALS [25–27,52,53], while EphA4 was identified to be the only known ALS modifier [30,31]. This implies that the binding of the MSP domain by HCV proteins will reduce the secretion of the MSP domain to function as an inhibitor of Eph receptors. This may lead to the similar syndromes triggered by other ALS pathogenesis, which include MSP mutations or/and reduced inhibition of Eph receptors. Indeed, ALS-like syndrome has been observed on the patient with chronic hepatitis C [32,54]. However, so far, only the interaction of the MSP domain with EphA4 has been biophysically characterized [27]. Here, we further characterized the interaction of the MSP domain with five more Eph receptors, namely EphA2, EphA5, EphA6, EphA7 and EphA8. The results reveal that EphA2 and EphA5 have higher affinity than EphA4, despite having the similar binding interfaces on the MSP domain. Our present results provide biophysical clues for further functional assessment about whether the HCV infection may additionally affect the signal pathways associated with EphA2 and EphA5.

In summary, for the first time we have successfully deciphered that the HCV NS5B utilizes its C-linker to bind the MSP domain of the human VAPB to form a dynamic complex. This finding implies that NS5B C-linker achieves dual roles by a conformational/dynamic switch between the folded state acting as an enzymatic auto-inhibitor, and disordered state ready for dynamically binding the host factor VAPB to initiate RNA synthesis. The identification that EphA2 and EphA5 bind the MSP domain with higher affinity than EphA4 offers clues for further addressing whether other than inducing ALS-like syndrome, the HCV infection might also trigger pathological consequences associated with signalling pathways mediated by EphA2 and EphA5.

## Materials and Methods

### DNA cloning

The DNA fragments encoding the human VAPB(1–195), MSP(1–125) and CCD(152–195) domains were amplified from HeLa cell cDNA library with designed primers as previously described [22]. All the VAPB DNAs were cloned into a modified pET32a vector (Novagen). DNA fragments encoding NS5B proteins were generated from HCV genotype 1b strain S1 (GenBank-AF356827.1). DNA encoding NS5B(1–570) was cloned into pET32a and pET22b expression vectors with N- and C-terminal His-tag respectively, while NS5B(1–531) was cloned into pET32a. The DNA fragment for the C-linker over residues 531–570 was amplified and cloned into pGEX-4T1 vector.

DNA fragments encoding the ephrin binding domain of Eph receptors (EphA2, EphA3, EphA4, EphA5, EphA6, EphA7, EphA8 receptor) were cloned into pET 32a (Novagen) as we previously described [26,27,31,33–38].

### Expression and purification of VAPB and NS5B proteins

After the sequences were confirmed by DNA sequencing analysis, plasmids were transformed into *Escherichia coli* BL21 (DE3) star (Invitrogen) competent cells. For the expression of recombinant protein, transformed cells with respective plasmids were grown in Luria-Bertani (LB) medium with 100 μg/ml ampicillin at 37°C to reach the absorbance of 0.6 at 600 nm and subsequently induced with respective optimized IPTG concentrations.

All the VAPB proteins were expressed and purified as we previously described [26]. For purifying NS5B proteins, harvested cells were resuspended and lysed by sonication in lysis buffer (50 mM Tris, 500 mM NaCl, 10% glycerol, 20 mM imidazole, 10 mM 2-mercaptoethanol, pH 7.5) containing protease inhibitor cocktail (Roche). His-tagged NS5B proteins were purified by Ni^2+^-affinity chromatography (Qiagen) while GST-fused NS5B(531–570) was purified by affinity chromatography with glutathione-Sepharose 4B beads (Pharmacia Biotech) under native conditions. Further purification of His-tagged proteins was achieved with Heparin Sepharose HiTrap HP column (GE healthcare). GST-tagged NS5B(531–570) was cleaved with thrombin and then the released C-linker peptide was purified by HPLC on a reverse-phase C18 column (Vydac).

Plasmids containing the ligand binding domain of 6 Eph receptors were transformed into *Escherichia coli* Rosetta-gami (DE3) cells (Novagen). Transformed cells were grown in LB media and protein was purified under native conditions using affinity chromatography followed by thrombin cleavage to remove the His-tag. Cleaved Eph proteins were further purified with HPLC size exclusion chromatography using HiLoad 16/60 Superdex 200 column (GE healthcare).

The production of the isotope-labeled VAPB and NS5B domain/fragment proteins for NMR studies followed a similar procedure except that the bacteria were grown in M9 medium with the addition of (^15^NH_4_)_2_SO_4_ for ^15^N-labeling, or (^15^NH_4_)_2_SO_4_/^13^C-glucose for ^15^N/^13^C-labeling. The purity of all protein samples was checked by the SDS-PAGE gel and their molecular weights were verified by a Voyager STR matrix-assisted laser desorption ionization time-of-flight-mass spectrometer (Applied Biosystems). The concentration of protein samples was determined by the spectroscopic method in the presence of denaturant [26].

### CD and NMR experiments

All far-CD experiments were carried out in a Jasco J-810 spectropolarimeter (Jasco Corporation, Tokyo, Japan) at 25°C as we described previously [26] at a protein concentration of 20 μM in 2 mM phosphate buffer (pH 6.8). Data from five independent scans were added and averaged.

All NMR samples were prepared in 10 mM phosphate buffer at pH 6.8. NMR data were collected at 25°C on an 800-MHz Bruker Avance spectrometer equipped with a shielded cryoprobe. Protein samples at a concentration of 100 μM were used for ^1^H one-dimensional or ^1^H-^15^N NMR two-dimensional HSQC characterization.

### NMR HSQC titrations

For NMR titration experiments, two-dimensional ^1^H-^15^N HSQC spectra of ^15^N- labeled VAPB proteins were acquired at a protein concentration of 50 μM in the absence or presence of the unlabeled NS5B/Eph proteins at different molar ratios in 10 mM phosphate buffer at pH 6.8. More specifically, for characterizing the interactions of the MSP domain with the NS5B C-linker peptide or human Eph receptors, a series of HSQC spectra of the ^15^N-labeled MSP domain at a protein concentration of 50 μM were collected in 10 mM phosphate buffer at pH 6.8, with gradual addition of the C-linker peptide or Eph receptors with a ratio increment of 0.5 until the shift of HSQC peaks were mostly saturated [26]. By superimposing the HSQC spectra at different molar ratios, the shifted or disappeared HSQC peaks could be identified and further assigned to the corresponding residues based on NMR assignments of VAPB domains we previously published [25–27]. The degree of perturbation was reflected by an integrated chemical shift difference (CSD) calculated by the formula ((Δ^1^H)^2^+(Δ^15^N)^2^/5)^1/2^ [26,33]. The calculated CSD tracings at various different molar ratios were fitted by using one binding site model [26,33] to obtain residue-specific dissociation constants (Kd), which are summarized in Table 1.
